# Sphingosine 1-phosphate Stimulates Insulin Secretion and Improves Cell Survival by Blocking Voltage-dependent K^+^ Channels in β Cells

**DOI:** 10.3389/fphar.2021.683674

**Published:** 2021-07-12

**Authors:** Zhihong Liu, Huanhuan Yang, Linping Zhi, Huan Xue, Zhihong Lu, Yanli Zhao, Lijuan Cui, Tao Liu, Shouan Ren, Peifeng He, Yunfeng Liu, Yi Zhang

**Affiliations:** ^1^ Department of Pharmacology, Shanxi Medical University, Taiyuan, China; ^2^ Department of Respiratory and Critical Care Medicine, First Hospital of Shanxi Medical University, Shanxi Medical University, Taiyuan, China; ^3^ Key Laboratory of Cellular Physiology, Ministry of Education, Shanxi Medical University, Taiyuan, China; ^4^ Department of Emergency Medicine, First Hospital of Shanxi Medical University, Shanxi Medical University, Taiyuan, China; ^5^ School of Management, Shanxi Medical University, Taiyuan, China; ^6^ Department of Endocrinology, First Hospital of Shanxi Medical University, Shanxi Medical University, Taiyuan, China

**Keywords:** sphingosine 1-phosphate, insulin secretion, β cell, voltage-dependent potassium channels, type 2 diabetes

## Abstract

Recent studies suggest that Sphingosine 1-phosphate (S1P) plays an important role in regulating glucose metabolism in type 2 diabetes. However, its effects and mechanisms of promoting insulin secretion remain largely unknown. Here, we found that S1P treatment decreased blood glucose level and increased insulin secretion in C57BL/6 mice. Our results further showed that S1P promoted insulin secretion in a glucose-dependent manner. This stimulatory effect of S1P appeared to be irrelevant to cyclic adenosine monophosphate signaling. Voltage-clamp recordings showed that S1P did not influence voltage-dependent Ca^2+^ channels, but significantly blocked voltage-dependent potassium (Kv) channels, which could be reversed by inhibition of phospholipase C (PLC) and protein kinase C (PKC). Calcium imaging revealed that S1P increased intracellular Ca^2+^ levels, mainly by promoting Ca^2+^ influx, rather than mobilizing intracellular Ca^2+^ stores. In addition, inhibition of PLC and PKC suppressed S1P-induced insulin secretion. Collectively, these results suggest that the effects of S1P on glucose-stimulated insulin secretion (GSIS) depend on the inhibition of Kv channels *via* the PLC/PKC signaling pathway in pancreatic β cells. Further, S1P improved β cell survival; this effect was also associated with Kv channel inhibition. This work thus provides new insights into the mechanisms whereby S1P regulates β cell function in diabetes.

## Introduction

The study and development of medication to safely increase insulin secretion is of great significance for the treatment of type 2 diabetes. Sphingosine 1-phosphate (S1P) is a phosphorylated sphingosine metabolite that regulates various cellular functions, from cell differentiation to apoptosis (Olivera, et al., 1993; Hla, 2001) and has been found to exert beneficial effects on several human diseases including organ transplantation, multiple sclerosis, cancer and neuroinflammatory disorders (Brinkmann, 2007). Accumulating evidence indicates that S1P has an antidiabetic effect. It has been reported that S1P regulated glucose metabolism by decreasing glycaemia and improving glucose tolerance in diabetic mice (He, et al., 2019). Previous research has shown that S1P administration could improve β-cell functions, increase insulin secretion and reduce β-cell apoptosis (Haass, et al., 2015; Hahn, et al., 2017; Shimizu, et al., 2000; Laychock, et al., 2006). Furthermore, recent clinical data have revealed significantly elevated plasma levels of S1P in type 2 diabetes, which may act as a compensatory protective mechanism in the disease (Randriamboavonjy, et al., 2009; Tong, et al., 2014). So far, as a potential therapeutic target for various diseases, S1P signaling has attracted extensive attention, including for its therapeutic implications in diabetes (Chaudhry, et al., 2017; Lewis, et al., 2016; Liu, et al., 2019).

It is now clear that most effects of S1P occur through the activation of its five specific G-protein-coupled receptors on the plasma membrane (Hla, 2001). Recently many studies have focused on the effects of S1P and its receptors, however study on S1P-induced insulin secretion is little, and its signaling mechanism is still largely unclear. In addition, insulin release is orchestrated by complex ion channel electrical activity (Zhang, et al., 2017). During these processes, the closure of adenosine triphosphate (ATP)-sensitive K^+^ channels initiates membrane depolarization at high glucose concentrations, resulting in the activation of voltage-dependent Ca^2+^ channels (VDCCs), followed by an increase in intracellular Ca^2+^ concentrations ([Ca^2+^]i) *via* Ca^2+^ influx, ultimately stimulating insulin secretion. Meanwhile, activation of voltage-dependent K^+^ (Kv) channels promotes action potential repolarization, leading to VDCC closure, and limiting the free Ca^2+^ inflow (MacDonald, et al., 2003). Therefore, blocking Kv channels prolongs the action potential duration (APD) and enhances glucose-stimulated insulin secretion (GSIS) (Herrington, et al., 2006; Jacobson, et al., 2007). However, the electrophysiological mechanisms and characteristics of S1P-induced insulin secretion remain largely unknown.

In this study, we investigated the effects of S1P on β-cell insulin secretion and the underlying electrophysiological mechanisms, and the blood glucose response to S1P treatment in mice. Furthermore, we explored the cellular signaling pathways related to these effects.

## Materials and Methods

### Animals

Adult male Wistar rats (weighing 180–220 g) and 6-wk-old male C57BL/6 mice were supplied by the Animal Facility Center of Shanxi Medical University (Taiyuan, China). The rats and mice were housed in a pathogen-free facility at constant temperature (25 ± 3°C) with a 12 h light/dark cycle and free access to granular food and water. All experimental protocols involving animals were performed in accordance with the ethical guidelines for animal research of Shanxi Medical University and were approved by its Animal Care and Use Committee. The investigation conforms with the Guide for Care and Use of Laboratory Animals published by the US National Institutes of Health (8th edition, 2011).

### Isolation and Culture of Islets and Cells

Pancreatic islets and β cells were obtained from rats that had been anesthetized and then euthanized, as described previously (Gao, et al., 2016). Briefly, islets were separated by injecting collagenase P solution (1 mg/ml; Roche, Indianapolis, IN, United States), then digested at 37°C and subjected to density gradient centrifugation using Histopaque-1077 (Sigma-Aldrich, St. Louis, MO, United States). Single β cells were obtained from islets by dispase Ⅱ (Roche) digestion and seeded on small slides pretreated with cell adherent reagent (Applygen Technologies Inc., Beijing, China). Isolated rat islets and β cells were cultured in RPMI-1640 (Hyclone, South Logan, UT, United States) medium containing 10% fetal bovine serum (FBS; Gibco, Grand Island, NY, United States), in a humidified atmosphere of 5% CO_2_ at 37°C.

### Cell Line Culture

INS-1 β cells (Fenghuishengwu, Changsha, China) were cultured in RPMI-1640 medium supplemented with 10% FBS, 100 U/ml penicillin and 100 µg/ml streptomycin, 10 mM HEPES, 2 mM L-glutamine, 1 mM sodium-pyruvate, and 50 mM β-mercaptoethanol. Cells were incubated at 37°C in a humidified atmosphere with 5% CO_2_.

Chinese hamster ovary (CHO) cells (Fenghuishengwu) were stably transfected with Kv2.1 by adenoviral gene transfer technology, and were maintained in high-glucose Dulbecco’s Modified Eagle’s Medium (Hyclone) at 37°C in a humidified atmosphere with 5% CO_2_. These cells were passaged every 2–3 days by brief trypsin-ethylenediaminetetraacetic acid treatment. Cells were separated from their culture flask 24–48 h before experiments and were transferred to small coverslips or a plates.

### Insulin Secretion Assays

Rat pancreatic islets were preincubated for 30 min in Krebs-Ringer bicarbonate-HEPES (KRBH) buffer (128.8 mM NaCl, 4.8 mM KCl, 2 mM CaCl_2_, 1.2 mM KH_2_PO_4_, 1.2 mM MgSO_4_, and 5 mM NaHCO_3_, 10 mM HEPES, and 2% bovine serum albumin; pH 7.4) containing 2.8 mM glucose at 37°C. Then, KRBH buffer containing 2.8 or 16.7 mM glucose and different concentrations of S1P (Sigma-Aldrich) was added, and cells were incubated for another 30 min at 37°C. Later, the supernatants were collected and acute insulin secretion was detected using an insulin radioimmunoassay kit (Beijing North Biotechnology Institute Co., Beijing, China).

### Electrophysiological Experiments

Rat pancreatic β cells were cultured on small coverslips in RPMI-1640 medium at 37°C for 48 h or more, prior to experiments. The resistance of the patch pipettes filled with pipette solution (described below) ranged from 4 to 7 MΩ. Channel activity variations of β cells were recorded by whole-cell patch-clamp technology, using an EPC-10 amplifier and PULSE software (HEKA, Lambrecht, Germany) at room temperature (22–25°C). Islet β cells were identified by cell capacitance values >7 picofarads (pF) (Gopel, et al., 1999).

For Kv-current recording, patch pipettes were filled with intracellular solution containing 0.3 mM Mg-ATP, 10 mM NaCl, 1 mM MgCl_2_, and 0.05 mM ethylene glycol-bis(β-aminoethyl ether)-N,N,N′,N′-tetraacetic acid (EGTA), 140 mM KCl, and 10 mM HEPES and adjusted to pH 7.3 with KOH. β cells were voltage-clamped at a holding potential of −70 mV and shifted to test potentials from −70 to +80 mV in 10 mV steps every 400 ms. The extracellular solution contained 141.9 mM NaCl, 5.6 mM KCl, 1.2 mM MgCl_2_, 11.1 mM glucose, and 5 mM HEPES, and was adjusted to pH 7.4 with NaOH. The results of recorded Kv currents are shown as current densities (in picoamperes (pA)/pF).

To record VDCC currents, β cells were clamped to a holding potential of −70 mV, and shifted to test potentials from −50 to +30 mV in 10 mV steps every 50 ms. For Ca^2+^ current measurements, the intracellular solution contained 120 mM CsCl, 20 mM tetraethylammonium chloride (TEA; Sigma-Aldrich), and 5 mM Mg-ATP, 1 mM MgCl_2_, 0.05 mM EGTA, and 10 mM HEPES, and was adjusted to pH 7.3 with CsOH. The extracellular solution consisted of 100 mM NaCl, 20 mM TEA, 20 mM BaCl_2_, 4 mM CsCl, 1 mM MgCl_2_, 5 mM HEPES, and 3 mM glucose, and was adjusted to pH 7.4 with NaOH.

In the current-clamp pattern, action potentials were recorded by applying 4 ms pulses of a 150 pA current. APD was defined as the time from action potential initiation to the time that the cell membrane potential returned to within 10 mV of the resting membrane potential.

### Measurement of Cyclic Adenosine Monophosphate (cAMP) Production

Rat islets were incubated at 37°C for 1 h in KRBH solution containing 16.7 mM glucose, 500 µM isobutyl-1-methylxanthine (IBMX; Cayman, Ann Arbor, MI, United States), and 100 µM 4-(3-butoxy-4-methoxybenzyl) imidazolidin-2-one (Ro20-1724; Sigma-Aldrich). IBMX and Ro20-1724 are broad-range phosphodiesterase inhibitors that prevent cAMP degradation in the samples. S1P and forskolin (Cayman) were added to the solutions. Then, islets were lysed with an ultrasonic cell pulverizer (Xinzhi Biotechnology Co., Ningbo, China) and the total cAMP contents were detected using a radioimmunoassay method (Beijing North Biotechnology Institute Co.).

### Calcium Imaging

Fluorescent indicator Fura-2 AM (Dojindo Laboratories, Kumamoto, Japan) was used to measure [Ca^2+^]_i_. Freshly isolated rat pancreatic β cells were cultured on small coverslips coated with cellular adhesion reagent in RPMI-1640 medium at 37°C. The cells were incubated in KRBH buffer with 2 μM Fura-2 AM at 37°C for 30 min, and washed twice with KRBH buffer containing 2.8 mM glucose. Then, the β cells were continuously perfused with KRBH solution containing 16.7 mM glucose and different doses of S1P. Fluorescence ratio values were measured at excitation wavelengths of 380 and 340 nm and an emission wavelength of 510 nm, using an IX71 inverted microscope (Olympus Life Science, Tokyo, Japan) and MetaFluor Fluorescence Ratio Imaging Software 7.8 (Molecular Devices, Sunnyvale, CA, United States). Changes in fluorescence intensity values (F340/F380) were used to represent changes in [Ca^2+^]_i_. Average values 30 s before and after the peak of F340/F380 were used to compare differences between various treatments.

### Cell Viability Assay

Cell viability was measured by Cell Counting Kit-8 (CCK-8; Dojindo Laboratories) assay. INS-1 β cells and CHO-Kv2.1 cells were seeded in 96-well plates at a density of 3 × 10^4^ per well. After 24 h culture, the cells were added to palmitic acid (PA, 400 μM; Sigma-Aldrich), high-concentration glucose (30 mM), and S1P (10 µM), and cultured for another 24 h. Then, 10 μL CCK-8 solution was added to each well, and cells were incubated at 37°C for 2 h. Absorbance at 450 nm was detected using an ELISA reader (BioTek, Winooski, VT, United States). Cell viability was calculated as the ratios of the optical density values of each group and the control group.

### OGTT Evaluation of Drug Administration *in vivo*

At the age of 8 wk, C57BL/6 mice were given intraperitoneal injection of S1P (80 μg/kg, dissolved in 0.3 M NaOH and diluted 1:10 with PBS) (Fettel, et al., 2019) or vehicle, and waiting for 10 min to OGTT evaluation. For OGTT, mice were fasted for overnight (16 h) and fed with 2 g/kg glucose by gavage, then blood sample were gathered from the tail vein at indicated times. The blood glucose levels were assessed at baseline (0 min) and 15, 30, 60, 90, and 120 min using a Freestyle Optium Neo Glucometer (Abbott Laboratories, Chicago, IL, United States). The serum insulin levels were measured by Mercodia Mouse Insulin ELISA kit (stock number 10-1247-01; Uppsala, Sweden).

### Statistical Analysis

All experimental data are expressed as the mean ± SEM. Statistical analyses were performed using unpaired two-tailed Student’s t-test or one-way analysis of variance ANOVA, using SigmaPlot version 12.5 (Systat Software, San Jose, CA, United States) or Prism 8 (GraphPad Software, San Diego, CA, United States). Data from patch-clamp experiments were analyzed using PulseFit (HEKA) and Igor Pro 6.1 software (WaveMetrics Inc., Lake Oswego, OR, United States), and *p* < 0.05 was considered statistically significant.

## Results

### S1P Decreases Blood Glucose Levels by Increasing Plasma Insulin Levels *in vivo*

**FIGURE 1:**
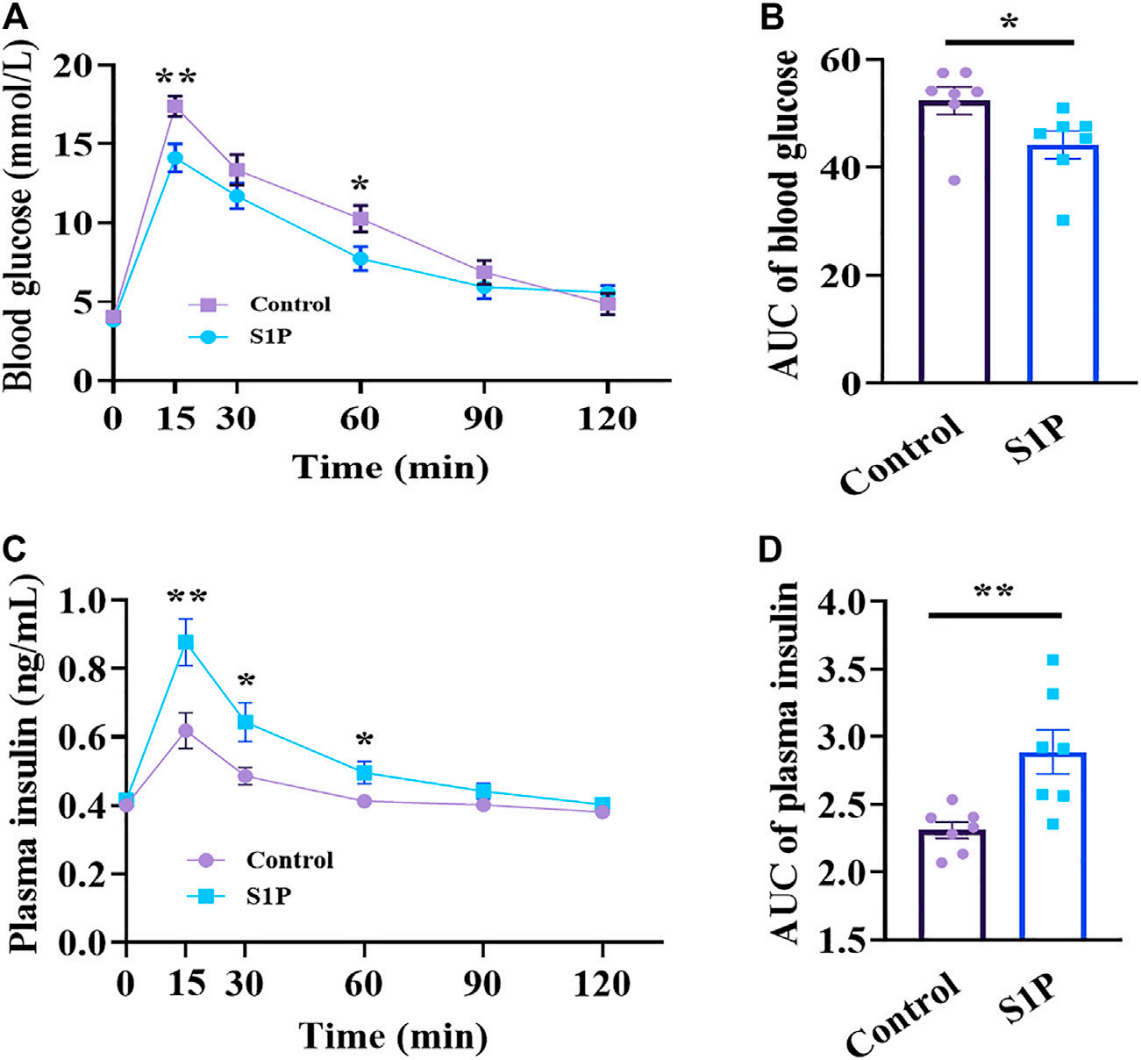
Effects of sphingosine 1-phosphate (S1P) treatment on blood glucose and plasma insulin *in vivo*. The 8-wk-old C57BL/6 mice were given intraperitoneal injection of S1P (80 μg/kg) or vehicle. After 10 min, the mice were fed with glucose load (2 g/kg) followed by determination of blood glucose level **(A)** and plasma insulin concentration **(C)** at the indicated time points. The AUC values of blood glucose **(B)** and insulin levels **(D)** during the OGTT were compared, respectively (*n* = 7). *p < 0.05, **p < 0.01 vs control group treated with the vehicle at each time point; by unpaired two-tailed Student’s t test. The results represent by the mean ± SEM.

We evaluated whether S1P induces glucose-lowering effect in 8-wk-old C57BL/6 mice following glucose administration *via* OGTT. The glucose-lowering effect of S1P was observed at 15 and 60 min *via* OGTT (Figure 1A), and significant decrease of glycemic level of S1P treatment was found using the area under the curve (AUC) compared with that of control group (Figure 1B). Furthermore, the plasma insulin levels of the S1P-treated group were obvious increase than those in the control group at 15, 30 and 60 min (Figure 1C), and the AUC between the two groups were also significant differences (Figure 1D). Therefore, these results show that the glucose-lowering effect of S1P was accompanied by the increase of plasma insulin levels, suggesting that S1P acts as an insulin secretagogue and exhibits glucose-lowering effects *in vivo*.

### S1P Potentiates GSIS, but Reduces Intracellular cAMP Production in Rat Pancreatic Islets

**FIGURE 2:**
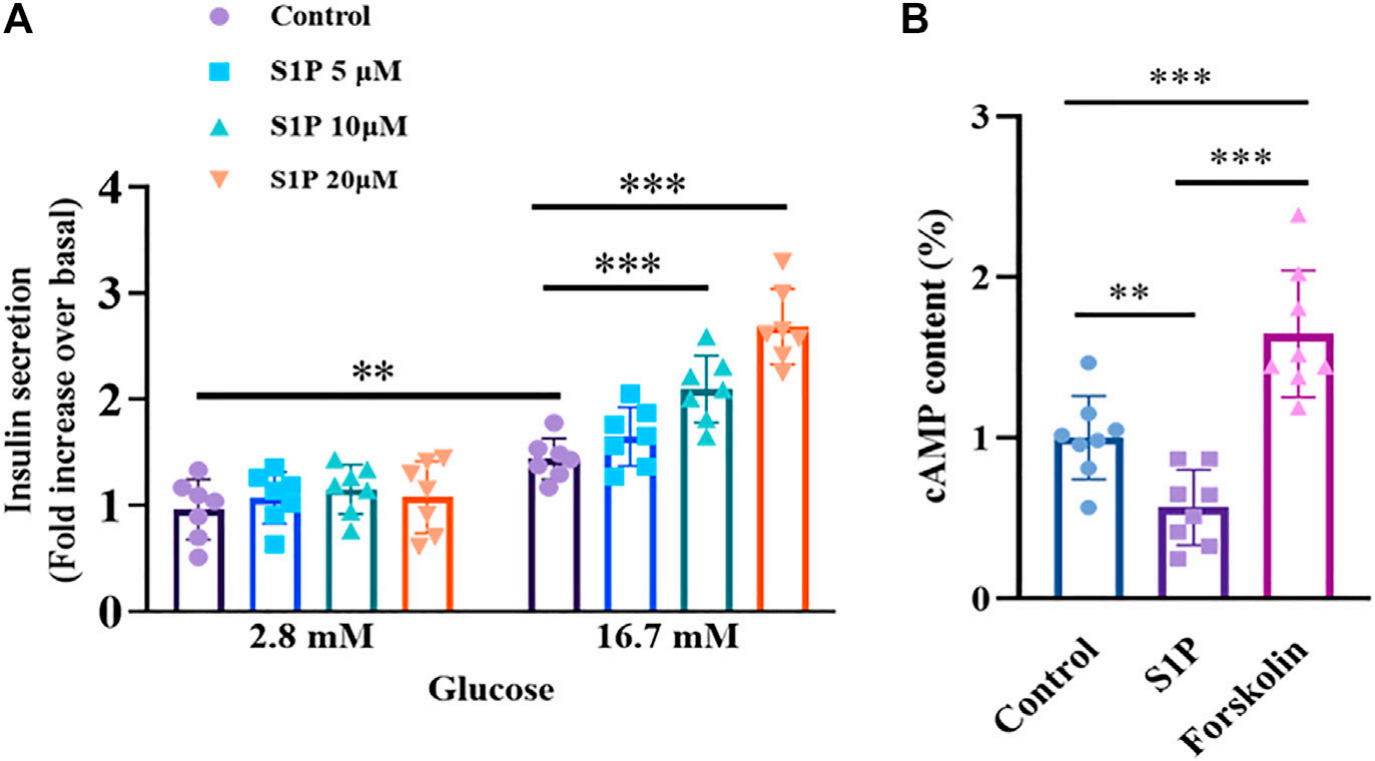
Effects of sphingosine 1-phosphate (S1P) on glucose-stimulated insulin secretion and intracellular cAMP production in rat islets. **(A)** Induction of insulin secretion by S1P under low- and high-glucose conditions (2.8 and 16.7 mM, respectively). The insulin secretion of each group was normalized by that of the control group with 2.8 mM glucose (*n* = 7). **(B)** Effect of S1P (10 μM) on rat islet cAMP levels. Forskolin (10 μM) was used as positive control. Intracellular cAMP levels were normalized by the control group (*n* = 8). **p < 0.01, ***p < 0.001; by one-way ANOVA. The results represent by the mean ± SEM.

We further examined the effects of S1P on insulin secretion from isolated rat islets in the presence of 2.8 or 16.7 mM glucose. As illustrated in Figure 2A, S1P had no effect on insulin secretion under low-glucose (2.8 mM) conditions. However, under high-concentration glucose (16.7 mM), S1P (10 and 20 µM) increased insulin secretion from the pancreatic islets, suggesting that S1P induced insulin secretion in a glucose-dependent manner. Based on these results, we selected 10 µM S1P for subsequent experiments.

Since intracellular cAMP is regarded as an important physiological amplifier of GSIS in pancreatic β cells (Furman, et al., 2010). We next tested whether S1P-induced insulin secretion was associated with increased cAMP contents. Notably, as shown in Figure 2B, a decreased in cAMP production, not an increase was observed following treatment with S1P (10 µM) under 16.7 mM glucose.

### S1P Increases [Ca^2+^]_i_ Concentration, but Does Not Affect VDCCs in Pancreatic β Cells

**FIGURE 3:**
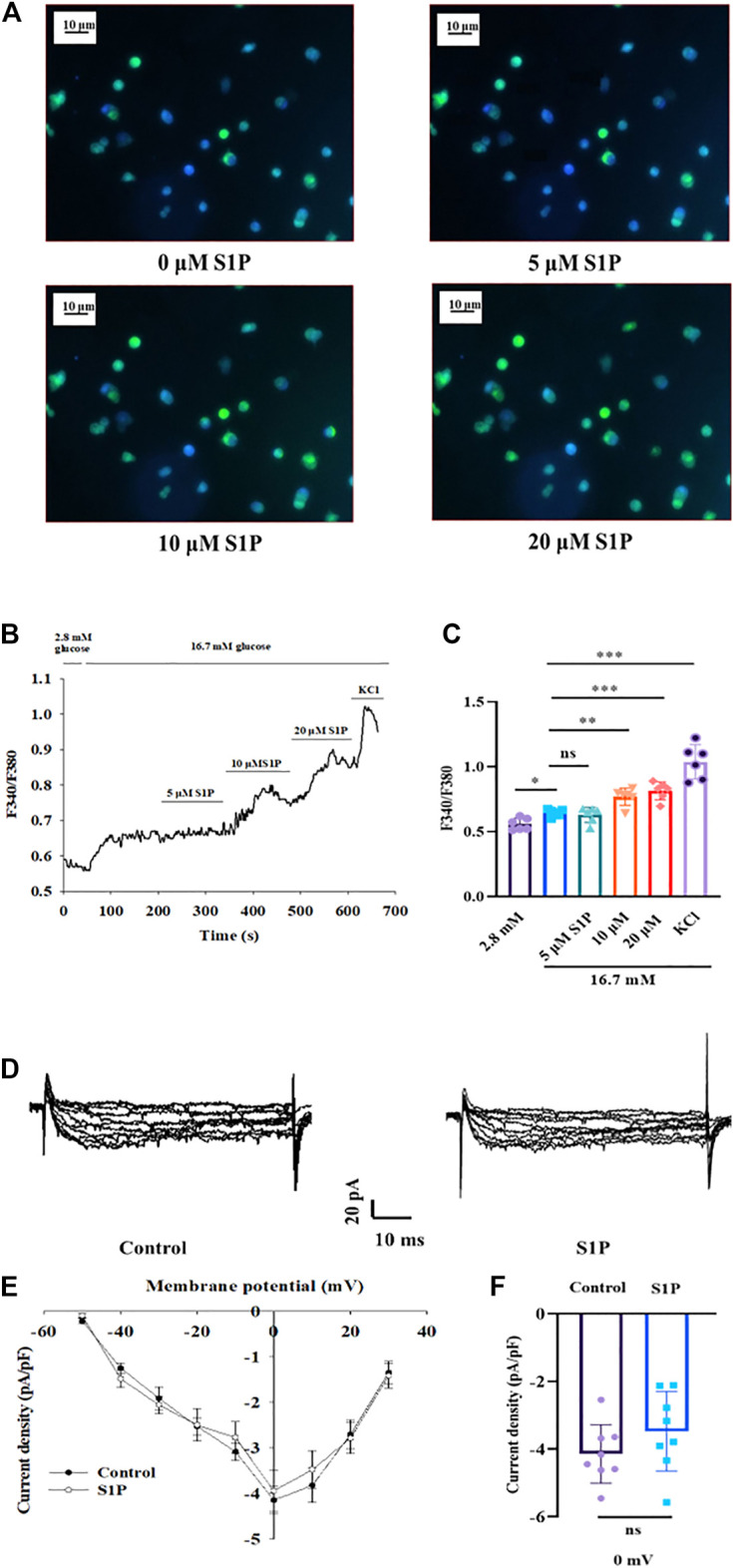
Effects of sphingosine 1-phosphate (S1P) on [Ca^2+^]_i_ and voltage-dependent Ca^2+^ channel (VDCC) activation in pancreatic β cells. **(A)** Photomicrograph showing changes in fluorescence of β cells after treatment with different S1P doses. Blue represents β cells stained with Fura-2 AM. Green represents the increase in fluorescence density of Fura-2 AM staining. **(B)** Changes in F340/F380 after treatment with different doses of S1P in 16.7 mM glucose, which reflects changes in [Ca^2+^]_i_. **(C)** Summary of the mean F340/F380 values after treatment with different doses of S1P (*n* = 6). *p < 0.05, **p < 0.01, ***p < 0.001; ns, not significant; by one-way ANOVA. **(D)** Representative current traces of VDCC recorded in the presence or absence of S1P (10 μM). **(E)** Current-voltage relationship curves of VDCCs in β cells. **(F)** Summary of the mean current densities recorded at 0 mV depolarization (*n* = 8). Statistical differences between two groups (with or without S1P) were compared using an unpaired two-tailed Student’s *t* test. The results represent by the mean ± SEM.

Elevation of the [Ca^2+^]_i_ level has been found to be vital for insulin secretion by β cells (Klec, et al., 2019). We next explored the effects of S1P on [Ca^2+^]_i_ by applying calcium imaging to detect changes in fluorescence intensity. S1P (at 10 and 20 µM) enhanced the fluorescence intensity of β cells under 16.7 mM glucose in a dose-dependent manner (Figures 3A–C), indicating that S1P induced an increase in [Ca^2+^]_i_ in β cells.

To verify whether this increase in [Ca^2+^]_i_ by S1P was related to activation of VDCCs, the whole-cell voltage-clamp technique was applied. The current-voltage relationship curves revealed that S1P did not influence inward Ca^2+^ current densities, relative to those of control group (Figures 3D–F).

### S1P Elevates [Ca^2+^]_i_ Mainly by Extracellular Ca^2+^ Influx, Not by Releasing Intracellular Ca^2+^ Stores

**FIGURE 4:**
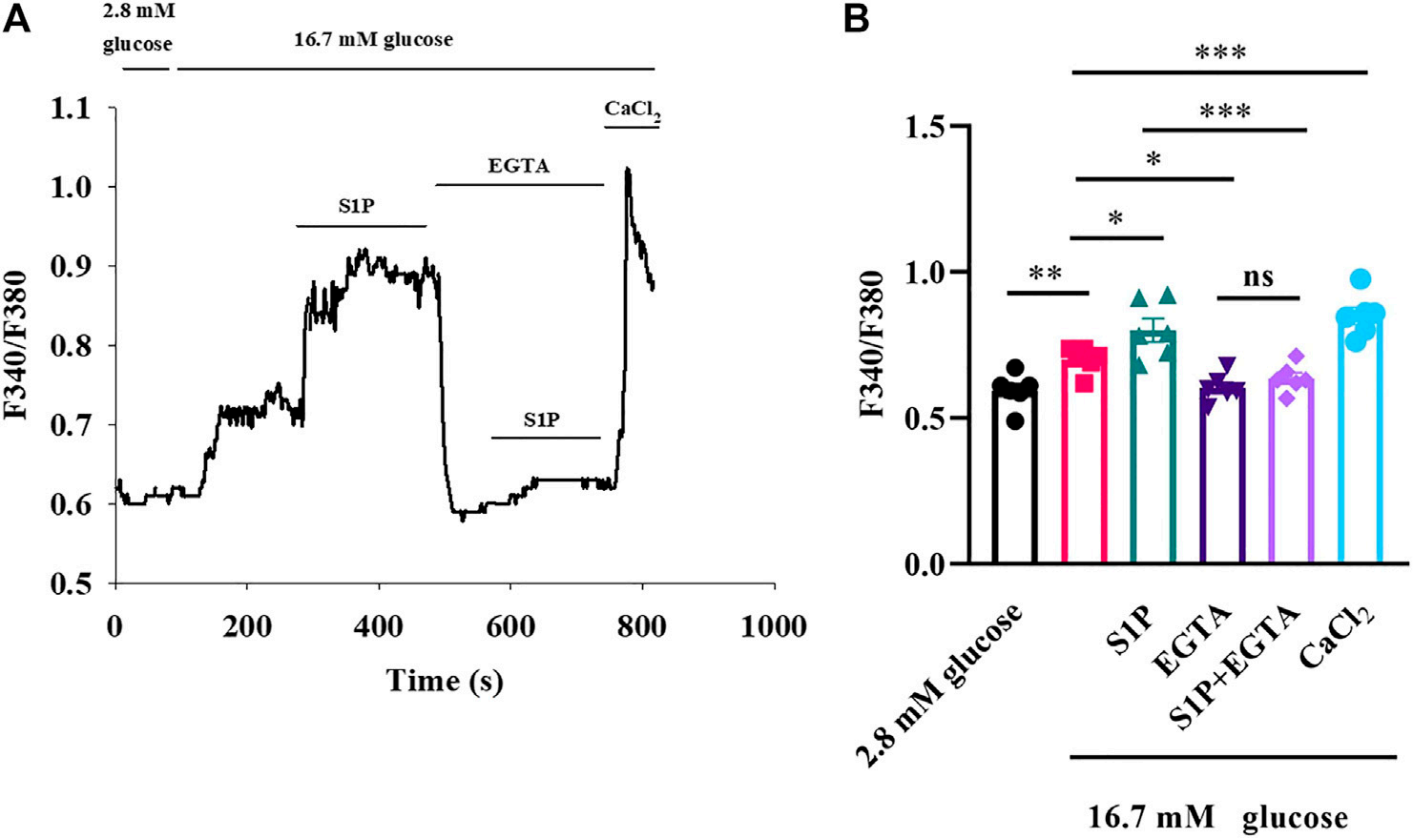
Sphingosine 1-phosphate (S1P) affected [Ca^2+^]_i_ by promoting Ca^2+^ influx rather than releasing intracellular Ca^2+^ stores. **(A)** Changes in F340/F380 values induced by S1P in the presence and absence of EGTA (2.5 mM). EGTA concentrations were sufficient to chelate the calcium present in the KRBH buffer (2 mM). Extracellular Ca^2+^ using CaCl_2_ (5 mM) was added to produce a final concentration of 5 mM of free Ca^2+^ at the end of the experiment. **(B)** Summary of the mean F340/F380 values with S1P (10 μM) treatments in the presence or absence of EGTA (*n* = 6). *p < 0.05, **p < 0.01, ***p < 0.001; ns, not significant; by one-way ANOVA. The results represent by the mean ± SEM.

As S1P did not increase the [Ca^2+^]_i_ by altering VDCC, we investigated whether the increased [Ca^2+^]_i_ originated from the intracellular Ca^2+^ stores. We measured changes in [Ca^2+^]_i_ concentrations after treatment with the membrane-impermeable Ca^2+^ chelator EGTA. Fluorescence calcium imaging showed that S1P increased the [Ca^2+^]_i_ level; however, applying EGTA substantially reversed the increase in [Ca^2+^]_i_ that occurred in response to S1P treatment (Figure 4). Notably, EGTA treatment did not completely eliminate the fluorescence signal induced by S1P. Although Ca^2+^ influx was the major contributor to the rise in [Ca^2+^]_i_ induced by S1P, some release from internal Ca^2+^ stores may also occur.

### S1P Prolongs the APD and Inhibits Kv Channels in Pancreatic β Cells

**FIGURE 5:**
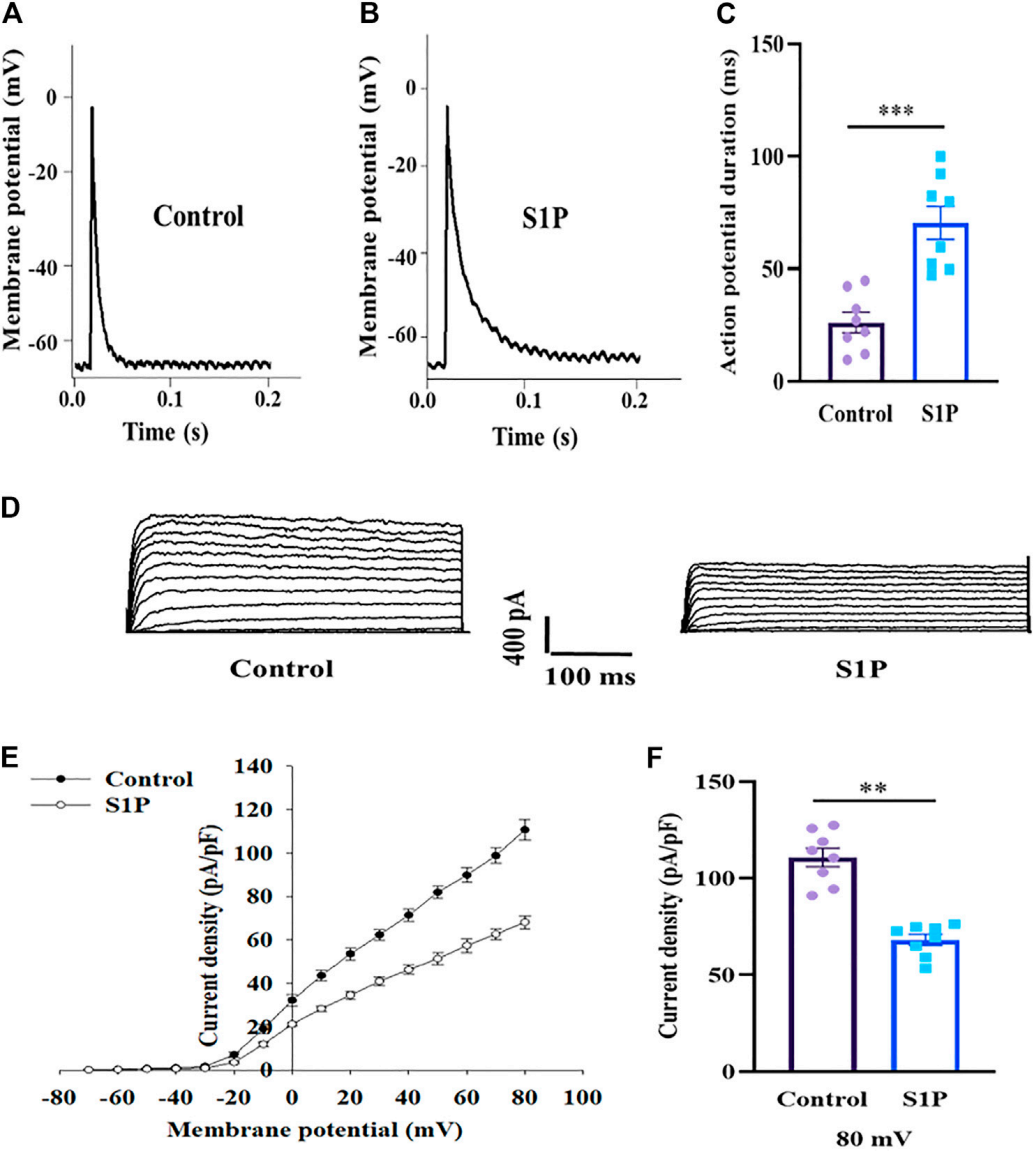
Pancreatic β cells treated by sphingosine 1-phosphate (S1P) exhibited lengthened action potential duration (APD) and lower Kv currents. **(A**,**B)** Action potential waveforms of β cells treated without **(A)** and with **(B)** S1P (10 μM). **(C)** Summary of the mean APDs (*n* = 8). **(D)** Representative current traces recorded in the absence and presence of S1P (10 μM). **(E)** Current-voltage relationship curves of β cell Kv channels. **(F)** Summary of the mean current densities of Kv channels at 80 mV depolarization (*n* = 8). **p < 0.01, ***p < 0.001; by unpaired two-tailed Student’s *t* test. The results represent by the mean ± SEM.

Action potentials play an important role in the stimulus–secretion coupling. We thus investigated whether action potentials were affected by S1P, using current-clamp mode. S1P significantly lengthened the APD compared to that observed in untreated cells (Figures 5A–C).

Prolonged APD can result from either increased depolarizing currents (e.g., voltage-dependent Ca^2+^ currents) or decreased repolarizing currents (e.g., voltage-dependent K^+^ currents). As VDCCs were unaffected by S1P, we measured outward K^+^ currents using the whole-cell voltage-clamp technique. S1P significantly attenuated outward K^+^ currents, reducing the Kv-current densities of β cells relative to those of control cells (Figures 5D–F).

### S1P Influences Kv Currents by Phospholipase C (PLC)/Protein Kinase C (PKC) Signaling Rather Than Directly Inhibition

**FIGURE 6:**
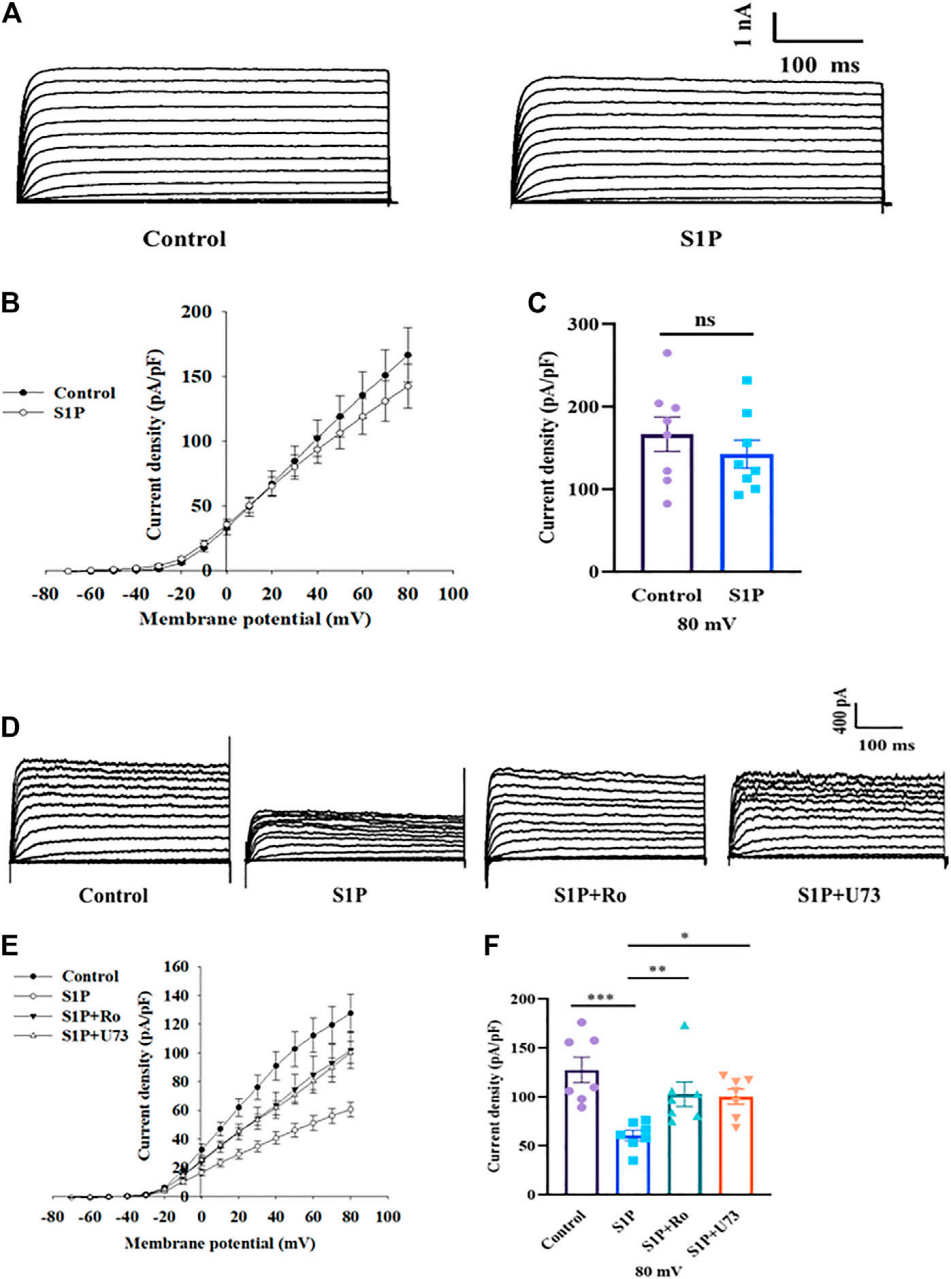
Sphingosine 1-phosphate (S1P) had no direct effects on Kv2.1 channels but regulated them by modulating phospholipase C and protein kinase C (PLC/PKC) signaling pathway. **(A)** Representative current traces in Chinese hamster ovary (CHO)-Kv2.1 cells recorded in the absence and presence of S1P (10 μM). **(B)** Current-voltage relationship curves of Kv2.1 channels from CHO-Kv2.1 cells. **(C)** Summary of the mean current densities of Kv2.1 channels recorded at 80 mV depolarization (*n* = 8; ns, not significant; by unpaired two-tailed Student’s *t* test). **(D)** Representative current traces recorded after β cells were treated with S1P (10 μM) in the presence of U73122 (U73, 10 μM) or Ro 31-8220 (Ro, 10 μM). **(E)** Current-voltage relationship curves of Kv channels under different treatment conditions. **(F)** Summary of the mean current densities of Kv channels recorded at 80 mV depolarization (*n* = 7). *p < 0.05, **p < 0.01, ***p < 0.001; by one-way ANOVA. The results represent by the mean ± SEM.

We next sought to determine how S1P inhibits Kv channels. Kv2.1 channels are the major Kv channel subtype in rat β cells and play an important role in insulin secretion (MacDonald, et al., 2002; Roe, et al., 1996). We overexpressed Kv2.1 channels in CHO cells and studied the effects of S1P, using a voltage-clamp experiment. The current-voltage relationship curves reveal that S1P did not directly inhibit Kv2.1 currents in CHO-Kv2.1 cells (Figures 6A–C).

Based on our previous results (Gao, et al., 2016), we explored whether the PLC/PKC pathway participates in the regulation of Kv channels by S1P using a voltage-clamp method. Pancreatic β cells were preincubated with either Ro 31-8220, a PKC inhibitor, or U73122, a PLC inhibitor. Thereafter, Kv currents in response to S1P were recorded using voltage-clamp technology. Both Ro 31-8220 and U73122 abolished S1P-induced inhibition of K^+^ currents (Figures 6D–F).

### S1P Augments GSIS *via* the PLC/PKC Pathway

**FIGURE 7:**
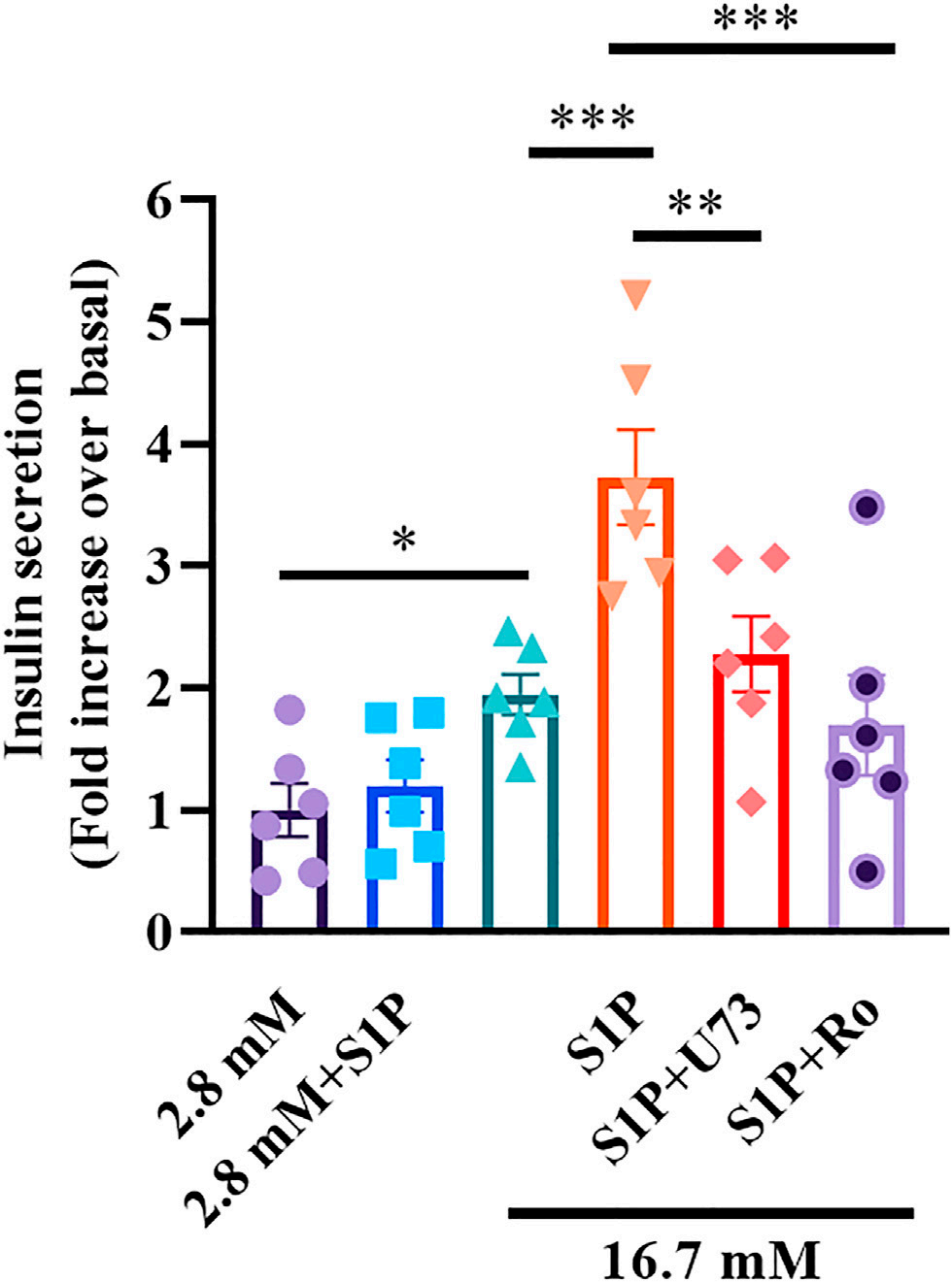
Sphingosine 1-phosphate (S1P) potentiated insulin secretion through the phospholipase C and protein kinase C (PLC/PKC) pathway. Effect of S1P on insulin secretion under 16.7 mM glucose conditions in the presence of S1P (10 μM), U73122 (U73, 10 μM), and Ro 31-8220 (Ro, 10 μM). U73 and Ro eliminated the insulinotropic effect of S1P (*n* = 6). *p < 0.05, **p < 0.01, ***p < 0.001; by one-way ANOVA. The results represent by the mean ± SEM.

As S1P inhibited Kv channels *via* the PLC/PKC pathway, we examined whether the PLC/PKC pathway was also involved in S1P-induced GSIS. As expected, the insulin secretion assay results showed that S1P treatment significantly enhanced GSIS. Moreover, U73122 or Ro 31-8220 eliminated this effect (Figure 7), indicating that the PLC/PKC pathway is also linked with GSIS induction by S1P.

### S1P Improves the Cell Viability of INS-1 Cells, but Not of CHO-Kv2.1 Cells, Treated With High-concentration Glucose and Palmitic Acid

**FIGURE 8:**
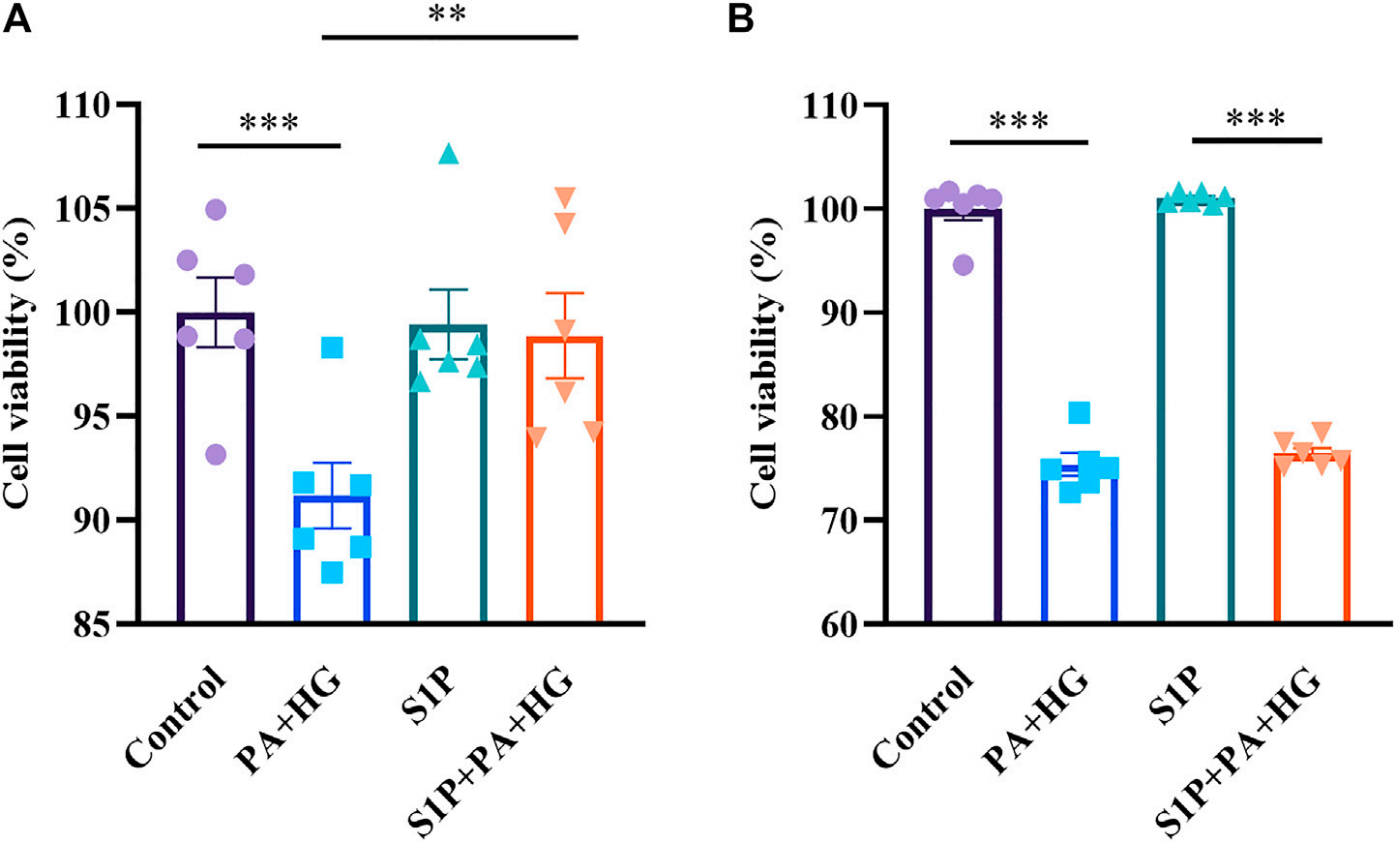
Effects of sphingosine 1-phosphate (S1P) on the cell viability of INS-1 cells and Chinese hamster ovary (CHO)-Kv2.1 cells. INS-1 cells or CHO-Kv2.1 cells were incubated with different treatments for 24 h, and viability was measured *via* the CCK8 assay. **(A)** S1P (10 μM) improved the viability of INS-1 cells treated with palmitic acid and high-concentration glucose (PA+HG, *n* = 6; **p < 0.01, ***p < 0.001; by one-way ANOVA). **(B)** S1P (10 μM) had no effect on the viability of CHO-Kv2.1 cells treated with palmitic acid and high-concentration glucose (PA+HG, *n* = 6; ***p < 0.001; by one-way ANOVA). The cell viability of each group was normalized by that of the control group. The results represent by the mean ± SEM.

The Kv channels, including Kv2.1, have been reported as important regulators of membrane potential and proliferation (Urrego, et al., 2014). It has been reported that the Kv2.1 channel is involved in programmed cell death and that Kv2.1 inhibition can prevent apoptosis (Jiao, et al., 2007). Hence, We further compared the difference of survival in INS-1 cells and CHO-Kv2.1 cells because of the different effects of S1P on Kv channels in both cells. The results showed S1P significantly improved the cell viability of INS-1 cells after treatment with high-concentration glucose and palmitic acid (Figure 8A), but did not affect that of CHO-Kv2.1 cells exposed to the same treatment (Figure 8B), suggesting that S1P exerted an influence on cell survival *via* inhibition of Kv channels.

## Discussion

S1P, a phosphorylated sphingosine metabolite, has been shown to exert important roles in various pathophysiological processes, including immune responses, angiogenesis, cell differentiation, apoptosis, inflammation, and cancer (Hatoum, et al., 2017; Aoki, et al., 2016; Maceyka, et al., 2012). Recent studies reported that S1P is beneficial for glucose regulation through improving insulin secretion, inhibiting β-cell apoptosis and reducing inflammation (Shimizu, et al., 2000; Cantrell Stanford, et al., 2012; Ng, et al., 2017). It has been reported that the levels of plasma S1P are as low as several nM to 100 nM, however, circulating platelets abundantly stores S1P and activated platelets may release S1P into the extracellular environment in response to physiological agonists such as thrombin, resulting in an obvious increase of local S1P concentrations, reaching the level of μM and influencing insulin secretion (Shimizu, et al., 2000). However, there are few studies on S1P-induced insulin secretion, and its underlying mechanism is still largely unclear. Although Most effects of S1P depend on its five G-protein-coupled receptor (GPCR) subtypes, other studies reported that S1P can act intracellularly as a second messenger (Laychock, et al., 2006; Hahn, et al., 2017), and also directly uptake into cell by S1P transporters (Goto, et al., 2021). In addition, as S1P biological effects are mediated by GPCR composed of five subtypes (S1PR1-5), their cellular effects are highly complex because each receptor subtype could signal *via* multiple G proteins signaling pathway. For such reasons, we did not attempt to identify the receptor subtypes linked with effect on S1P induced insulin secretion in β cells in this study, further research is needed in our next work. In this study, we mainly focused on the effect of S1P itself on insulin secretion and the related electrophysiological mechanisms. Our results showed that S1P potentiated insulin secretion and might act as an insulin secretagogue. This study further exhibited a novel mechanism whereby S1P regulated insulin secretion through inhibition of K_V_ channels.

Most insulin secretagogues in clinical treatment for controlling glycemia, such as sulfonylureas, increase insulin secretion in a glucose-independent manner, which means they may stimulate insulin secretion even under low-glucose conditions; they therefore carry a high risk of hypoglycemia incidents (Hayward, et al., 1997), which is a common and even fatal complications in diabetes treatment (Frier, et al., 2011). In the present study, it’s worth noting that S1P-induced insulin secretion is glucose-dependent, which imply a reduced risk of hypoglycemia. In addition, glucose-dependent insulin secretion is considered to be a more physiologically regulated process, and even may protect β cells (Islam, 2020). Thence, S1P may be a promising drug candidate for the treatment of type 2 diabetes mellitus.

The electrical activity of several ion channel participates in the functional regulation of β cells. Among them, sulfonylureas block K_ATP_ channels, which results in depolarization of the membrane that causes calcium influx and increases [Ca^2+^]_i_, which thereby triggers insulin secretion. The sulfonamide diazoxide produces the opposite effect: it activates K_ATP_ channels, resulting in a decreased insulin secretion (Quesada, et al., 1999; Trube, et al., 1986). In this study, we treated β cells with high concentration of glucose and glucose metabolism will close K_ATP_ channels. In addition, Kv channels play vital roles in β-cell membrane repolarization, and act as a negative regulator of insulin secretion (MacDonald, et al., 2002; Yang, et al., 2014). Tetraethylammonium (TEA), a non-selective delayed rectifier potassium channel blocker, blocks Kv channels and exerts regulating effects on cells (Yan, et al., 2019). Here, our data revealed that S1P strongly inhibited Kv channels and significantly extended the APD, finally effectively stimulating GSIS *via* this underlying mechanism. It has been reported that the Kv2.1−/− β-cells have current amplitudes that are only 17% that of controls (Jacobson, et al., 2007). Our results showed S1P produced an about 50% inhibition of the Kv channel current. Although other Kv channel subtypes other than Kv2.1 channels may also be suppressed, the inhibitory effect of S1P on Kv channel is mainly contributed by Kv2.1 channels.

The cAMP-dependent signaling pathway is an important pathway for promoting GSIS. Some insulinotropic hormones, such as glucagon-like peptide-1 (GLP-1) and gastric inhibitory polypeptide (GIP), enhanced insulin secretion through increased intracellular cAMP production (Tengholm, et al., 2017). Nevertheless, we and others have observed that S1P reduced cAMP contents (Shimizu, et al., 2000; Jongsma, et al., 2009; Johnstone, et al., 2005; Pierre, et al., 2004), suggesting that S1P appears to promote insulin secretion *via* a distinct mechanism other than cAMP signaling.

The PLC/PKC cascade is considered as another important signaling pathway that contributes to sustained and enhanced GSIS (Kalwat, et al., 2017; Trexler, et al., 2017). It is reported that cholecystokinin and acetylcholine have a positive impact on insulin secretion *via* the PLC/PKC pathway (Ghorbani, et al., 2019). In the present study, our findings reveal that blockade of PLC or PKC not only inhibited insulin secretion, but also reduced Kv currents, indicating that PLC/PKC signaling pathway was involved in the regulation of S1P on insulin secretion and Kv channel effects in β cells. The result was also supported by previous study that PKC inhibitors can eliminate GSIS induced by free fatty acids and rutin, a flavonoid substance (Kalwat, et al., 2017; Kappel, et al., 2013). Moreover, further studies on the effect of PKC on insulin secretion showed that PKC might play a role through inducing direct phosphorylation of critical exocytotic proteins and enhancing the calcium sensitivity of exocytosis (Trexler, et al., 2017). Nevertheless, our current findings provide a novel viewpoint, suggesting that PLC/PKC pathway mediates S1P-induced insulin secretion by regulating Kv channel activation in β cells.

Previous studies indicated that S1P increases [Ca^2+^]_i_ by mobilizing intracellular Ca^2+^ stores through the PLC/IP_3_-dependent pathway (Blaho, et al., 2014; Blom, et al., 2005). S1P-lyase overexpression evoked an attenuated calcium leak from the endoplasmic reticulum and a lowered cytosolic calcium level in rat islets and INS1E cells (Hahn, et al., 2017). In this study, we found that S1P increased [Ca^2+^]_i_ in pancreatic β cells. The elevation of [Ca^2+^]_i_ in β cells stimulates insulin vesicle exocytosis (Fridlyand, et al., 2013). However, our further results show that the S1P-stimulated [Ca^2+^]_i_ levels were reduced compared with those observed in Ca^2+^-containing KRB solution in the absence of extracellular Ca^2+^ (chelated by EGTA), suggesting S1P markedly increased [Ca^2+^]_i_ in pancreatic β cells, mainly through extracellular Ca^2^+ influx. And other reports support this notion that extracellular Ca^2+^ influx *via* L-VDCC is crucial for regulating [Ca^2+^]_i_ in β cells (Bokvist, et al., 1995; Hwang, et al., 2018). In our study, patch clamp results showed that the increase in [Ca^2+^]_i_ is related to the inhibition of Kv channels by S1P, which prolongs the duration of the action potential, indirectly increases voltage dependent Ca^2+^ influx, instead of directly affecting VDCC. Therefore, our results propose a different perspective from previous studies, that the elevation of [Ca^2+^]_i_ by S1P might be mostly due to Ca^2+^ influx rather than Ca^2+^ release from internal stores in β cells. Nevertheless, our results do not exclude the possibility of some Ca^2+^ release from internal Ca^2+^ stores. It is generally accepted that [Ca^2+^]_i_ concentration is tightly regulated by an interplay between extracellular Ca^2+^ influx and the inositol 1,4,5-trisphosphate (IP_3_)-mediated release of Ca^2+^ from intracellular stores (Barker, et al., 2015), and PLC signaling provide a connection between them and contributed to maintain the proper dynamics of [Ca^2+^]_i_ (Thore, et al., 2004).

In addition to the effects on insulin secretion, K^+^ efflux has been reported to affect the activation of intracellular caspases, indicating that Kv channels are involved in the process of cell survival (Valencia-Cruz, et al., 2009). Blockade of Kv channels increased the cell viability and prevented the apoptosis of cortical neurons stimulated by high-concentration glucose (Yan, et al., 2019). The Kv2.1 channel is considered to be a major Kv channel subtype in β cells (MacDonald, et al., 2002). Kv2.1 channel overexpression in INS-1 cells has been shown to promote apoptosis (Kim, et al., 2012), and downregulation of Kv2.1 reduced apoptosis in cerebellar neurons (Jiao, et al., 2007). In this study, we showed that S1P improve the survival of INS-1 cells mainly through regulation of Kv channels. This effect is consistent with another study (Zhou, et al., 2016), in which Kv channel inhibition efficiently promote GSIS and prevent apoptosis in β cells. Although previous study showed that high concentration of S1P (above 5 µM) acted intracellularly and caused cell viability loss in insulin-secreting INS-1E cells (Hahn, et al., 2017), other studies indicated that S1P (5 or 10 µM) improved the viability of β cells, and these protective effect attributed to the regulation of mitochondrial action and prohibitin protein expression (Hong, et al., 2018), and to the adenosine 5′-monophosphate-activated protein kinase (AMPK)-dependent pathway (Ng, et al., 2017), our findings suggest that Kv channel may also play an important role in β cell survival improved by S1P. In addition, we noted that the suppression of apoptosis produced by inhibiting the Kv channel involved a variety of downstream signaling pathways. Considering that these signaling pathways involved in cell protection are complex, additional studies are required to further clarify the relationship between the effects of S1P on Kv channels and its survival-promoting effects.

**FIGURE 9:**
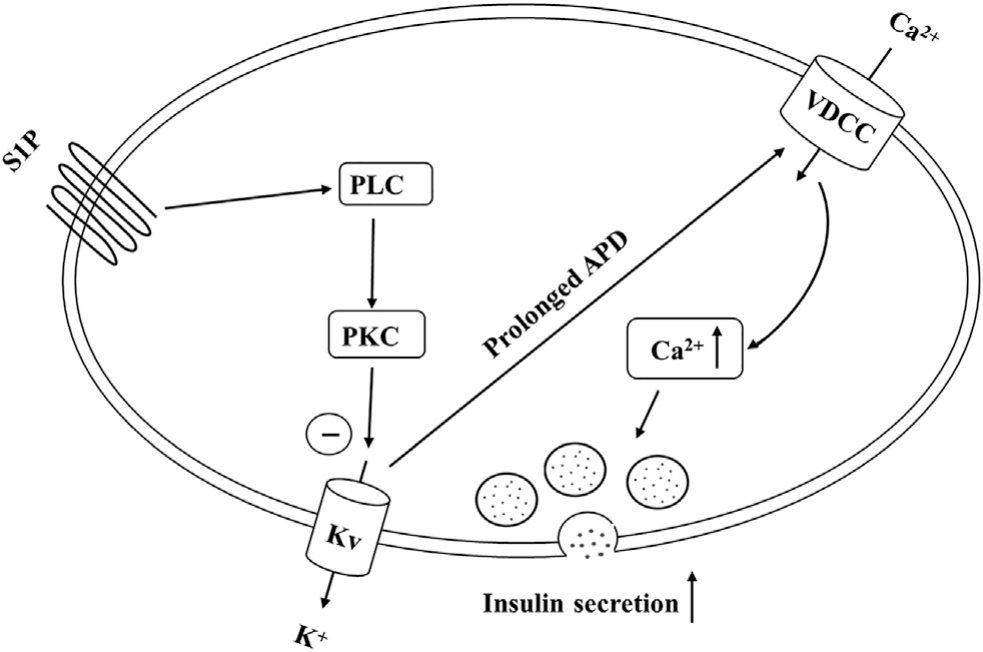
Schematic of our proposed molecular mechanism by which sphingosine 1-phosphate (S1P) induces insulin secretion from pancreatic β cells. S1P inhibits voltage-dependent potassium (Kv) currents by activating the PLC/PKC pathway (rather than the adenylate cyclase (AC)/cAMP pathway), and not by direct suppression, thereby prolonging action potential duration (APD). This promotes Ca^2+^ influx and increases the intracellular Ca^2+^ concentration, finally stimulating insulin secretion.

In summary, our findings demonstrate that S1P potentiates GSIS by activating PLC/PKC pathway, inhibiting Kv channels and increasing [Ca^2+^]_i_ in pancreatic β cells (Figure 9). Furthermore, S1P also promoted β-cell survival by inhibiting the Kv channels. Therefore, we propose that Kv channels play a key role in S1P-regulated β cell function. This is a novel insight into the mechanisms whereby S1P counteracts diabetes, which may have important implications for the prevention and management of type 2 diabetes.

## Data Availability

The original contributions presented in the study are included in the article, further inquiries can be directed to the corresponding authors.
